# Motor imagery and resistance training improve strength in older adults through distinct effects on agonist–antagonist coordination

**DOI:** 10.3389/fpsyg.2026.1817866

**Published:** 2026-04-20

**Authors:** Wan X. Yao, Bernadett Mamone, Yuntao Wu, Bo Y. Jiang, John Q. Zhang, Jianmin Guan, Guang H. Yue

**Affiliations:** 1 Department of Kinesiology, College for Health, Community and Policy, University of Texas at San Antonio, San Antonio, TX, United States; 2 Neurelis Inc., San Diego, CA, United States; 3 Beijing Rehabilitation Medicine Academy, Capital Medical University, Beijing, China; 4 Changchun Kuancheng District Centers for Disease Control and Prevention and Health Inspection Institute, Jilin, China; 5 Center for Mobility and Rehabilitation Engineering, Kessler Foundation, West Orange, NJ, United States; 6 Department of Physical Medicine and Rehabilitation, Rutgers New Jersey Medical School, Rutgers, The State University of New Jersey, Newark, NJ, United States

**Keywords:** aging, antagonist muscle activation, co-contraction, motor imagery training, neuromuscular control, older adults, rehabilitation, strength training

## Abstract

**Objective:**

Age-related declines in muscle strength are partly attributable to altered neural drive and agonist–antagonist coordination. This study aimed to evaluate the effects of motor imagery training (MIT) on maximal force production and neuromuscular activation patterns in healthy older adults, with conventional strength training (CST) included as a reference intervention.

**Methods:**

Thirty-two right-handed older adults were randomly assigned to an 8-week MIT (*n* = 12), CST (*n* = 12), or non-exercise control group (CTRL; *n* = 8). Twenty-seven participants completed the intervention (MIT: *n* = 11; CST: *n* = 10; CTRL: *n* = 6). Training was performed 5 sessions/week. Maximal voluntary isometric elbow flexion force and surface EMG activity of the biceps brachii (agonist) and triceps brachii (antagonist) were recorded pre- and post-intervention. Antagonist muscle co-activation (AMCA) and co-contraction index (CCI; antagonist-to-agonist EMG ratio) were quantified. Post-intervention values were analyzed using ANCOVA with baseline values as covariates. Exploratory within-subject correlation analyses were conducted to examine associations between changes in force and coordination metrics.

**Results:**

Both MIT and CST significantly increased maximal force compared with CTRL (*p* < 0.001), with no difference between training modalities. Both interventions significantly reduced CCI (*p* < 0.001), indicating improved relative agonist–antagonist coordination. In contrast, absolute antagonist muscle co-activation (AMCA) did not change significantly in either group. Exploratory analysis further revealed significantly greater post-intervention agonist EMG in both MIT and CST compared with CTRL. In addition, exploratory analyses revealed a significant negative association between changes in force and changes in CCI, whereas no significant association was observed between changes in force and AMCA.

**Conclusion:**

Both MIT and CST were associated with improvements in strength and coordination efficiency in older adults. Exploratory findings further suggest that strength gains may be more closely aligned with changes in relative agonist–antagonist coordination than with changes in absolute antagonist activation. Exploratory agonist EMG analysis further provides context suggesting that increased agonist activation may contribute, at least in part, to the observed coordination changes. These findings are consistent with a contribution of neural mechanisms, although direct neurophysiological measures are needed to confirm the underlying processes. Motor imagery training may therefore represent a viable low-load strategy for neuromuscular rehabilitation in aging populations.

## Introduction

Age-related declines in muscle strength are well-established and are among the strongest predictors of functional limitation and disability in older adults (Hunter et al., 2016; Manini, 2011). Although reductions in muscle mass contribute to this decline, neural factors—including diminished voluntary activation, altered motor unit recruitment, and impaired corticospinal drive—play a substantial role in age-related weakness (Clark, 2019; Enoka and Duchateau, 2017; Yue et al., 1999). Accordingly, interventions that target neural mechanisms, in addition to peripheral muscle adaptations, are of growing interest in physical and rehabilitation medicine.

Beyond force capacity, aging involves significant alterations in neuromuscular coordination. Older adults commonly exhibit increased antagonist muscle co-activation, often interpreted as a compensatory “stiffening strategy” to enhance joint stability amidst declining sensorimotor function (Hortobágyi and Devita, 2006; Lee et al., 2017; Narici et al., 2008; Toda et al., 2016). While these strategies may improve stability, they concurrently reduce movement efficiency and net torque production. Whether these entrenched coordination patterns are modifiable through different training modalities, especially those bypassing peripheral loading, remains a critical question in motor control.

Conventional strength training (CST) is widely regarded as the gold standard for improving muscle strength and physical function in aging populations (Arnold and Bautmans, 2014; Borde et al., 2015). Resistance-based training or CST enhances strength through combined peripheral and central adaptations, including increased motor unit recruitment, elevated firing rates, improved voluntary activation, and in some cases, reductions in antagonist co-activation (Carolan and Cafarelli, 1992; Enoka and Duchateau, 2017). These adaptations collectively improve force production and may enhance coordination efficiency. However, CST requires sufficient physical capacity and joint tolerance, which may limit feasibility in frail individuals such as older adults or those with pain, osteoarthritis, or mobility restrictions.

An emerging “top-down” approach is motor imagery training (MIT), a cognitive strategy in which individuals mentally rehearse a motor action without producing overt muscle activity (Jeannerod and Decety, 1995). MIT is task-specific and engages neural circuits that substantially overlap with those activated during actual movement execution (Park and Li, 2011). Experimental evidence indicates that MIT increases corticospinal excitability (Ranganathan et al., 2004; Yao et al., 2013) and enhances voluntary strength in both young and older adults (Reiser et al., 2011; Yue and Cole, 1992), with some studies suggesting relatively greater benefits in older populations (Liu et al., 2022). Additionally, MIT has been shown to induce bilateral transfer effects, further supporting its central neural basis (Land et al., 2016; Yao et al., 2023; Yue and Cole, 1992). Although MIT has been associated with improvements in force production and reductions in co-contraction index (CCI) (Yao et al., 2025), its influence on absolute antagonist muscle co-activation remains insufficiently characterized.

Importantly, antagonist muscle behavior can be quantified using distinct but complementary metrics. Antagonist muscle co-activation (AMCA) reflects the absolute magnitude of antagonist activation during agonist contraction, whereas the co-contraction index (CCI) represents the relative balance between agonist and antagonist activation. Distinguishing between these metrics allows for a more precise examination of whether training reduces absolute antagonist involvement or instead improves coordination efficiency through enhanced agonist activation and proportional scaling of muscle activity (Hortobágyi and DeVita, 2000).

Despite growing interest in neural-based interventions, direct comparisons between MIT and CST on force production and distinct coordination metrics in older adults are limited. Moreover, it remains unclear whether improvements in strength following MIT or CST are mediated by reductions in antagonist interference, enhanced agonist activation, or refinement of agonist–antagonist coupling.

Therefore, the primary aim of the present study was to evaluate the effects of an 8-week motor imagery training (MIT) program on maximal voluntary isometric elbow flexion force and neuromuscular coordination in older adults. A secondary objective was to compare outcomes between MIT and conventional strength training (CST), without formal equivalence testing.

Specifically, we examined changes in force output, antagonist muscle co-activation (AMCA), and co-contraction index (CCI) to assess whether training-related improvements were associated with alterations in absolute antagonist activation or changes in coordination efficiency. In addition, exploratory analyses were conducted to examine potential differences in neuromuscular adaptation patterns between a primarily centrally mediated intervention (MIT) and a combined neural–peripheral intervention (CST).

We hypothesized that both MIT and CST would increase maximal voluntary isometric elbow flexion force compared with a non-exercising control condition. We further hypothesized that MIT-related improvements would be associated with enhanced coordination efficiency, reflected by reductions in CCI, without substantial changes in absolute antagonist activation (AMCA). In contrast, CST was expected to influence both force production and neuromuscular activation patterns through combined neural and peripheral adaptations. These hypotheses were tested with an emphasis on group differences relative to control, with comparisons between MIT and CST interpreted as secondary.

## Research methods

### Ethics approval

The study protocol was approved by the Cleveland Clinic Institutional Review Board prior to the commencement of data collection and was conducted in accordance with the principles of the Declaration of Helsinki. All participants provided written informed consent before participation.

### Study design and overview

This study employed a randomized, controlled, parallel-group design to evaluate the effects of an 8-week motor imagery training (MIT) program and a conventional strength training (CST) program on upper-limb force production and neuromuscular coordination in older adults.

Primary outcome measures included:

- Maximal voluntary isometric elbow flexion force (maximal EF force)
- Antagonist muscle co-activation of the triceps brachii (AMCA)
- Co-contraction index (CCI), reflecting the relative balance between agonist and antagonist activation.

### Participants

Thirty-two right-handed older adults (mean age 74.4 ± 7.1 years; 23 females) were enrolled and randomly assigned to a motor imagery training group (MIT; *n* = 12), a conventional strength training group (CST; *n* = 12), or a no-exercise control group (CTRL; *n* = 8). Allocation ratios were determined a priori to prioritize statistical power for comparisons between the two active intervention groups, consistent with the mechanistic focus of the study and accepted approaches in randomized trial design (Dumville et al., 2006; Torgerson and Torgerson, 2008).

Hand dominance was determined using the Edinburgh Handedness Inventory (Oldfield, 1971). Participants were community-dwelling and free from neurological, musculoskeletal, or cardiovascular conditions affecting upper-limb function. Individuals with recent upper-limb injury, surgery, or pain limiting maximal effort were excluded.

Twenty-seven participants (age range: 65–85 years) completed the intervention phase (MIT: *n* = 11; CST: *n* = 10; and CTRL: *n* = 6). Only participants who completed both pre- and post-intervention assessments were included in the final analysis.

### Experimental procedure

#### Strength measurement

Maximal voluntary isometric elbow flexion (EF) force was assessed with participants seated. The left arm was positioned in the sagittal plane with approximately 10 ° shoulder abduction. The elbow was flexed to approximately 90 ° and supported at hip height, with the forearm in an intermediate position between pronation and supination. The wrist was secured in a cuff attached to an O-shaped metal frame connected to a force transducer (JR3, model 45E15A-U760-100L450, Woodland, CA, USA).

Participants performed five maximal voluntary contractions, each separated by sufficient rest. Prior to testing, participants completed several submaximal elbow flexion contractions as familiarization trials to ensure understanding of the task and minimize potential learning effects. Loud verbal encouragement was provided during maximal efforts to promote consistent exertion, and the highest force value across trials was used for analysis.

#### EMG recording, processing, and data analysis

Surface EMG was recorded from the biceps brachii muscle (BB; agonist) and the triceps brachii muscle (TB; antagonist) during maximal voluntary isometric elbow flexion trials. Bipolar surface electrodes (8-mm diameter) were placed over the muscle bellies according to standard anatomical guidelines.

EMG signals were amplified (500–5,000 ×), band-pass filtered (10 Hz−1 kHz) using a Neurodata Amplifier system (Model 15A54, Grass Instrument Co., Quincy, MA, USA), digitized at 2,000 Hz, and stored for offline analysis. EMG data were processed using Spike2 software (Cambridge Electronic Design, Cambridge, UK) and custom-written routines. Signals were full-wave rectified and averaged over a 1-s window corresponding to the peak force plateau.

To account for inter-individual variability, EMG amplitudes were normalized to the highest EMG value obtained across the five maximal voluntary contractions for each muscle. Antagonist muscle co-activation (AMCA) was defined as the normalized EMG activity of the triceps brachii during maximal voluntary isometric elbow flexion.

Neuromuscular coordination was further assessed using the co-contraction index (CCI), which quantifies the relative balance between antagonist and agonist activation during maximal voluntary contraction. CCI was calculated as:


$$\text{CCI }\left(\%\right)\text{=}\frac{{\text{EMG}}_{\text{TB}}\text{ during EF MVC}}{{\text{EMG}}_{\text{BB}}\text{ during EF MVC}}\text{ X 100}$$

Detailed descriptions of the force and EMG measurement procedures have been reported previously (Ranganathan et al., 2004).

### Interventions

#### Motor imagery training

Participants in the MIT group completed an 8-week training program consisting of 30-min sessions, 5 days per week. Participants were seated in an experimental chair with the left forearm positioned in a neutral orientation between supination and pronation, the elbow flexed at approximately 90 °, and the shoulder abducted approximately 10 °, consistent with the positioning used during strength testing.

Training emphasized first-person, kinesthetic motor imagery of maximal elbow flexion contractions. At the beginning of each session, participants received standardized instructions designed to facilitate vivid kinesthetic imagery. These instructions guided participants to focus on internal sensations associated with maximal effort, including progressive muscle contraction and force generation during elbow flexion.

Participants completed 3–5 supervised practice trials at the beginning of training sessions (5 trials during the first three sessions) to ensure task understanding. Following familiarization, participants performed 30 imagery trials (three sets of ten), with approximately 20 s rest between trials and 3 min rest between sets. Auditory cues were used to guide the timing of imagery contractions.

To monitor compliance and minimize unintended muscle activation, surface EMG electrodes were placed over the biceps brachii and triceps brachii muscles during selected sessions (once per week). Participants were instructed to avoid overt muscle contraction during imagery. All sessions were supervised to ensure adherence and consistency across participants.

#### Conventional strength training

Participants in the CST group completed an 8-week resistance training program targeting the elbow flexor muscles, with a schedule matched to the MIT group (30-min sessions, 5 days per week). Training consisted of repeated maximal or near-maximal isometric elbow flexion contractions performed using the same apparatus and joint positioning as in strength testing.

Each session included 30 contractions (three sets of ten), with approximately 20 s rest between contractions and 3 min rest between sets. Participants were instructed to generate maximal voluntary force during each contraction while maintaining proper positioning.

Training intensity was maintained at maximal or near-maximal effort throughout the intervention, and participants received standardized verbal encouragement to ensure consistent effort. The total number of sessions and overall training volume were matched to the MIT protocol. All sessions were supervised to ensure adherence and consistency across participants.

#### Control condition

Participants in the control group received no training intervention and were instructed to maintain their usual physical activity levels throughout the study period. In addition, to provide a time- and attention-matched condition, participants were asked to engage in quiet reading of their choice during the intervention sessions.

### Statistical analysis

Primary analyses were conducted using analysis of covariance (ANCOVA), with post-intervention values as the dependent variables, group (MIT, CST, CTRL) as the between-subjects factor, and baseline values as covariates. This approach was selected to account for baseline variability and is consistent with recommended methods for analyzing randomized studies with baseline and follow-up measurements (Vickers and Altman, 2001). These analyses were conducted for maximal elbow flexion force, co-contraction index (CCI), antagonist muscle co-activation (AMCA), and normalized agonist EMG (biceps brachii, BB).

When a significant main effect of group was observed, *post-hoc* pairwise comparisons were performed within the ANCOVA framework.

Effect sizes for ANCOVA were reported using partial eta squared (η^2^). Statistical significance was set at p <0.05.

In addition to primary analyses, exploratory within-subject change analyses were conducted. Change scores (Δ) were calculated as post-intervention minus pre-intervention values for maximal elbow flexion force, co-contraction index (CCI), and antagonist muscle co-activation (AMCA). Pearson correlations were used to assess associations between change in force and coordination metrics. These analyses were considered exploratory and hypothesis-generating.

### Data availability

The data associated with this study are not publicly available but are available from the corresponding author on request.

## Results

This study assessed pre- and post-intervention values of maximal elbow flexion (EF) force and neuromuscular coordination following motor imagery training (MIT) and conventional strength training (CST) relative to a control condition (CTRL). Significant group differences were observed for EF force and CCI, whereas no significant differences were found for AMCA. Detailed statistical analyses supporting these findings are presented below.

### Maximal EF force

An analysis of covariance (ANCOVA), adjusting for baseline values, revealed a significant main effect of group on post-intervention force, *F*_(2, 23)_ = 12.65, *p* < 0.001, partial η^2^ = 0.52. Baseline force was a significant covariate, *F*_(1, 23)_ = 442.16, *p* < 0.001, partial η^2^ = 0.95.

*Post-hoc* comparisons based on the ANCOVA model showed that both the MIT and CST groups demonstrated significantly greater force than the control group [MIT vs. CTRL: *F*_(1, 23)_ = 18.72, *p* < 0.001, partial η^2^ = 0.45; CST vs. CTRL: *F*_(1, 23)_ = 21.35, *p* < 0.001, partial η^2^ = 0.48]. No significant difference was observed between the MIT and CST groups [*F*_(1, 23)_ = 0.64, *p* = 0.43, partial η^2^ = 0.03].

Adjusted post-intervention force values were higher in both the MIT group [105.59, 95% CI [101.21, 109.97]] and CST group [109.28, 95% CI [104.70, 113.86]] compared with the control group [91.49, 95% CI [85.56, 97.41]].

**Figure 1:**
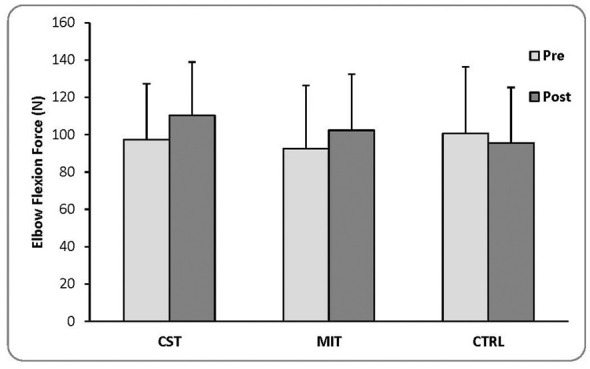
Pre- and post-intervention maximal elbow flexion (EF) force across groups (mean ± SD). Values are presented as mean ± standard deviation (SD) for the conventional strength training (CST), motor imagery training (MIT), and control (CTRL) groups. Statistical comparisons were performed using analysis of covariance (ANCOVA) with adjustment for baseline values. Both MIT and CST demonstrated significantly greater post-intervention force compared with CTRL, with no significant difference between MIT and CST.

Descriptive statistics (means ± SD) for pre- and post-intervention EF force are presented in Figure 1.

### Neuromuscular coordination

An analysis of covariance (ANCOVA), adjusting for baseline values, revealed a significant main effect of group on post-intervention CCI, *F*_(2, 23)_ = 9.84, *p* < 0.001, partial η^2^ = 0.46. Baseline CCI was a significant covariate, *F*_(1, 23)_ = 318.57, *p* < 0.001, partial η^2^ = 0.93.

*Post-hoc* comparisons based on the ANCOVA model showed that both the MIT and CST groups demonstrated significantly lower CCI than the control group [MIT vs. CTRL: *F*_(1, 23)_ = 11.92, *p* = 0.002, partial η^2^ = 0.34; CST vs. CTRL: *F*_(1, 23)_ = 18.47, *p* < 0.001, partial η^2^ = 0.45]. No significant difference was observed between the MIT and CST groups [*F*_(1, 23)_ = 2.11, *p* = 0.16, partial η^2^ = 0.08].

Adjusted CCI values were lower in both the MIT group [20.87, 95% CI [17.87, 23.88]] and CST group [18.17, 95% CI [15.03, 21.31]] compared with the control group [34.05, 95% CI [29.98, 38.12]].

**Figure 2:**
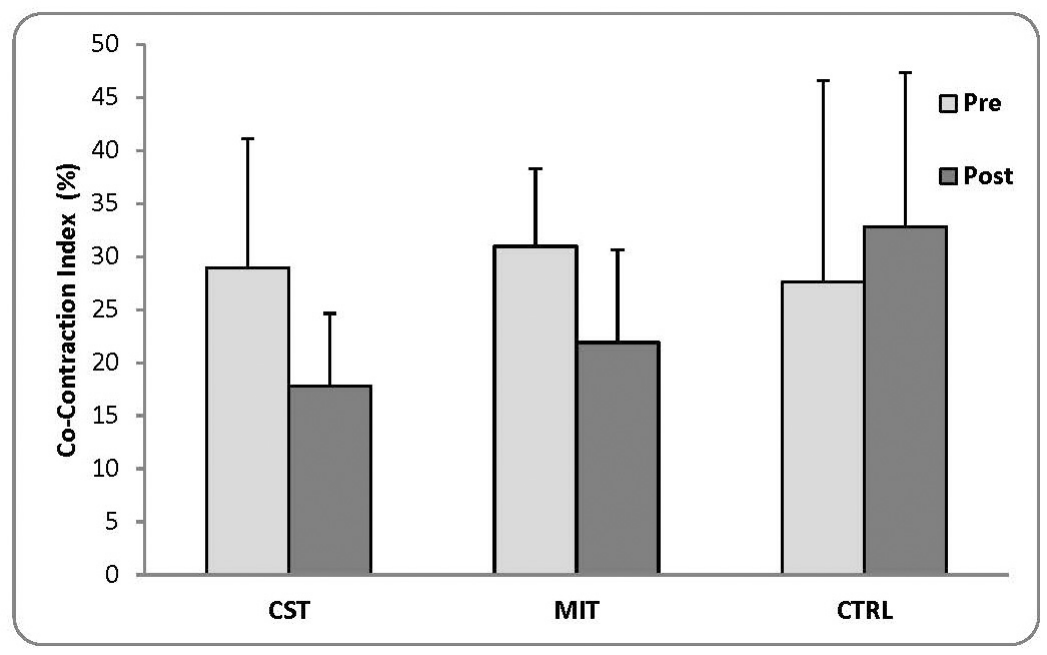
Pre- and post-intervention co-contraction index (CCI) across groups (mean ± SD). Values are presented as mean ± SD for CST, MIT, and CTRL. Statistical comparisons were conducted using ANCOVA with adjustment for baseline values. Both MIT and CST showed significantly lower post-intervention CCI compared with CTRL, with no significant difference between MIT and CST.

Descriptive statistics (means ± SD) for pre- and post-intervention CCI are presented in Figure 2.

An exploratory analysis of covariance (ANCOVA), adjusting for baseline values, revealed a significant main effect of group on post-intervention agonist EMG (BB), *F*_(2, 23)_ = 5.05, *p* = 0.015. Baseline BB EMG was a significant covariate, *F*_(1, 23)_ = 24.79, *p* < 0.001.

*Post-hoc* comparisons based on the ANCOVA model showed that both the MIT and CST groups demonstrated significantly greater post-intervention BB EMG than the control group (MIT vs. CTRL: *p* = 0.006; CST vs. CTRL: *p* = 0.015), whereas no significant difference was observed between the MIT and CST groups (*p* = 0.648).

An analysis of covariance (ANCOVA), adjusting for baseline values, revealed no significant main effect of group on post-intervention AMCA, *F*_(2, 23)_ = 0.39, *p* = 0.68, partial η^2^ = 0.03. Baseline AMCA was a significant covariate, *F*_(1, 23)_ = 210.47, *p* < 0.001, partial η^2^ = 0.90.

**Figure 3:**
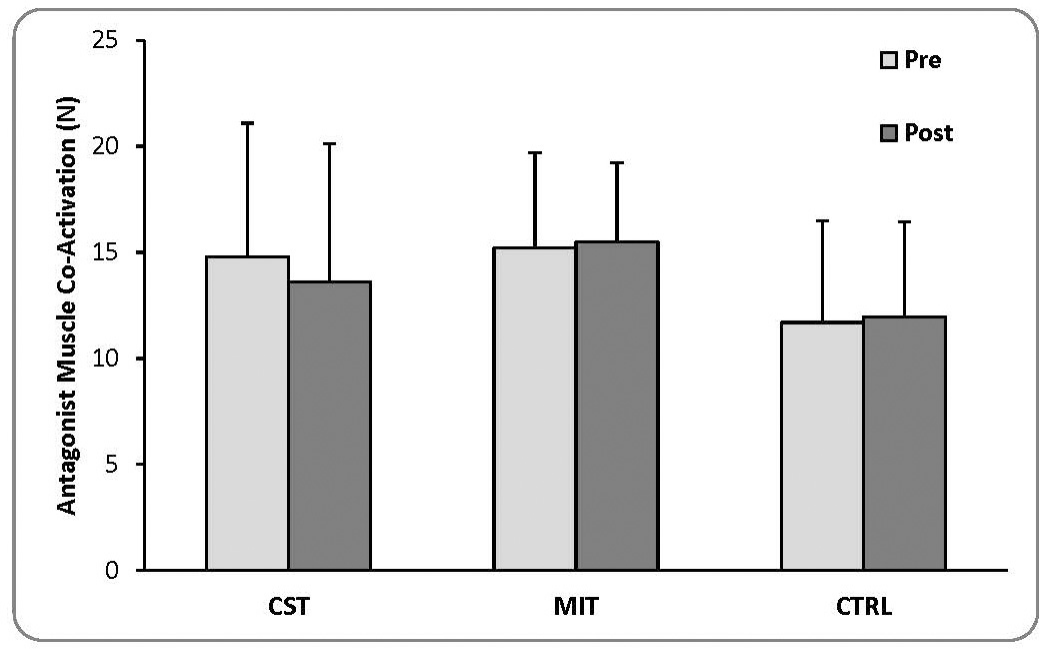
Pre- and post-intervention antagonist muscle co-activation (AMCA) across groups (mean ± SD). Values are presented as mean ± SD for CST, MIT, and CTRL. Statistical comparisons were performed using ANCOVA with adjustment for baseline values. No statistically significant group differences were observed.

Although no statistically significant differences were observed, a qualitative divergence in the direction of change was noted. The CST group showed a mean reduction in AMCA, whereas both the MIT and control groups showed slight mean increases, suggesting potentially distinct patterns of neuromuscular adaptation across interventions. Descriptive statistics are presented in Figure 3.

### Exploratory within-subject change analyses

Exploratory within-subject change analyses were conducted to further characterize the relationship between strength and neuromuscular coordination. Across all participants, a significant negative correlation was observed between ΔMVC and ΔCCI (*r* = −0.57, *p* = 0.002). In contrast, the association between ΔMVC and ΔAMCA was not statistically significant (*r* = −0.28, *p* = 0.158).

## Discussion

This study examined whether two mechanistically distinct training paradigms—motor imagery training (MIT) and conventional strength training (CST)—could modify upper-limb strength and neuromuscular coordination in older adults. The primary findings suggest that both paradigms elicited robust improvements in maximal force and significant reductions in co-contraction index (CCI). However, a key dissociation was observed in absolute antagonist behavior: while CST showed a mean reduction in antagonist muscle co-activation (AMCA), the MIT group exhibited a slight mean increase. Although these changes were not statistically significant, this divergence provides insight into potentially distinct neural strategies employed by mental vs. physical training to achieve functional gains.

The present findings should be interpreted with primary emphasis on the effects of MIT relative to the control condition, while comparisons between MIT and CST are considered secondary and exploratory.

### EF force gains following MIT and CST

Both MIT and CST produced significant improvements in maximal EF force relative to the control condition, with no significant difference between the two training modalities. These findings are consistent with previous work demonstrating that strength gains in older adults can be achieved through both physical and imagery-based interventions (Borde et al., 2015; Grgic et al., 2020; Jiang et al., 2016; Liu et al., 2022; Yao et al., 2025).

Whereas, CST is known to enhance force production through a combination of neural and peripheral adaptations, including increased motor unit recruitment, firing rate modulation, and muscle hypertrophy, MIT is thought to operate predominantly through central neural mechanisms, such as increased corticospinal excitability, enhanced motor planning, and improved voluntary drive.

The absence of a statistically significant difference in force gains between MIT and CST suggests that both interventions were associated with improvements in maximal force; however, this comparison should be interpreted cautiously, as the study was not designed to test equivalence. These findings are consistent with the possibility that central neural mechanisms may contribute to improvements in maximal force output, particularly in older adults with reduced neuromuscular efficiency. Accordingly, the results suggest that improvements in strength may be achieved with reduced physical loading under certain conditions, highlighting the potential of MIT as an alternative or complementary intervention for individuals with limited tolerance for high-intensity exercise.

### Neuromuscular coordination: relative vs. absolute antagonist behavior

The most novel aspect of the present findings lies in the dissociation between relative and absolute measures of antagonist involvement. Both MIT and CST were associated with significant reductions in CCI compared with the control condition (p <0.001), indicating improved relative coordination efficiency during maximal force production. However, neither intervention significantly altered AMCA.

This pattern highlights an important conceptual distinction: AMCA reflects the magnitude of antagonist activation, whereas CCI reflects how antagonist activation is scaled relative to agonist output. Because CCI represents a ratio of antagonist to agonist activation, reductions in CCI may arise from increased agonist activation, decreased antagonist activation, or both. To provide additional context for this ambiguity, exploratory analysis of agonist EMG was conducted. Both MIT and CST showed greater post-intervention BB EMG than the control group, with no difference between the two training groups. Although this additional analysis does not fully resolve the identifiability limitations of a ratio-based metric, it is consistent with the possibility that increased agonist activation contributed, at least in part, to the observed reduction in CCI.

To further examine the relationship between coordination metrics and strength changes, exploratory within-subject analyses were conducted. These analyses revealed a significant negative association between changes in maximal force and changes in CCI, whereas no significant association was observed between changes in maximal force and AMCA. Although these analyses were exploratory and do not establish causal mechanisms, this pattern is consistent with the interpretation that changes in relative agonist–antagonist coordination may be more closely aligned with strength changes than changes in absolute antagonist activation.

Accordingly, rather than indicating that training enhanced agonist activation or reduced antagonist activity directly, the findings are more conservatively interpreted as consistent with improvements in relative coordination efficiency. In other words, coordination efficiency might improve through changes in relative activation balance, not through elimination of antagonist involvement.

This finding contrasts with some lower-limb training studies reporting reductions in antagonist EMG activity following resistance training (Carolan and Cafarelli, 1992). The discrepancy may reflect limb- and task-specific control strategies. Upper-limb isometric tasks may rely less on postural stabilization demands and therefore exhibit different antagonist modulation patterns compared with dynamic lower-limb movements (Simoneau et al., 2006). These results suggest that improvements in neuromuscular performance do not uniformly require reductions in antagonist magnitude and may instead depend on optimization of activation ratios.

### Differential mechanisms underlying MIT and CST adaptations

Although MIT and CST produced behavioral outcomes, the underlying mechanisms are likely to differ. CST is known to induce adaptations at multiple levels of the neuromuscular system, including enhanced voluntary drive, altered motor unit recruitment patterns, improved spinal inhibitory modulation, and task-specific sensory feedback that refines agonist–antagonist interactions (Carolan and Cafarelli, 1992; Kido et al., 2004; Sale, 1986). These mechanisms provide a plausible basis for coordination improvements following resistance training.

In contrast, MIT appears to achieve similar functional improvements predominantly through top-down neural mechanisms, such as enhanced motor planning, increased corticospinal excitability, and improved central drive, without requiring peripheral loading or sensory feedback (Yao et al., 2013; Yue and Cole, 1992). The absence of significant AMCA modulation following MIT is consistent with this interpretation, as imagery-based training does not directly engage peripheral muscle-tendon or spinal reflex pathways.

Although the CST group showed a directional trend toward reduced AMCA and the MIT group did not, these differences were not statistically significant and should be interpreted cautiously. Importantly, the absence of statistically significant AMCA changes does not exclude modest antagonist modulation, particularly given the modest sample size. Nonetheless, the observed pattern is consistent with a possible mechanistic distinction between interventions. These findings suggest that MIT may primarily influence central aspects of motor control, whereas CST may additionally involve peripheral and spinal adaptations.

Collectively, these findings suggest that both MIT and CST were associated with improvements in strength and coordination efficiency; however, the underlying mechanisms may differ. In the absence of direct neurophysiological measures, these mechanistic interpretations should be considered inferential.

### Limitations

While the findings of this study provide meaningful insight into training-related neural adaptations, several limitations warrant consideration. First, the sample size was modest, particularly in the control group (*n* = 8). Although this allocation was preplanned to prioritize comparisons between the two active interventions (MIT and CST), the resulting unequal group sizes may have reduced statistical power to detect small-to-moderate effects, particularly for outcomes such as absolute antagonist muscle co-activation (AMCA). Accordingly, the absence of statistically significant differences in AMCA should not be interpreted as conclusive evidence that antagonist activation magnitude is unmodifiable, as modest antagonist modulation may not have been detectable given the sample size. The observed directional trends, especially within the CST group, warrant replication in larger samples.

Second, no direct neurophysiological measures (e.g., transcranial magnetic stimulation, H-reflex testing, or motor unit decomposition) were obtained. As a result, mechanistic interpretations regarding corticospinal excitability, spinal inhibition, or motor unit behavior remain inferential and grounded in established literature rather than direct physiological measurement.

Third, the interpretation of electromyographic indices such as the co-contraction index (CCI) is inherently limited, as changes in CCI may arise from multiple factors, including alterations in agonist activation, antagonist activation, or normalization procedures. In the absence of direct neurophysiological measures of neural drive or motor unit behavior, the mechanistic interpretation of these changes should be considered inferential.

Fourth, motor imagery ability was not formally assessed using standardized questionnaires (e.g., MIQ or KVIQ), which may limit interpretation of individual variability in training response.

Fifth, although a time- and attention-matched control condition was implemented through quiet reading, this condition does not fully control for non-specific factors such as expectancy, motivation, or engagement associated with active training interventions. In addition, repeated exposure to maximal voluntary contraction testing may have contributed to performance changes over time. These factors cannot be fully excluded and should be considered when interpreting the results.

Finally, sex-stratified analyses were not conducted due to unequal sex distribution across intervention groups. Given known sex-related differences in neuromuscular control in older adults, future studies with balanced group allocation are needed to determine whether MIT and CST effects differ by sex.

Despite these limitations, the controlled intervention design, standardized protocols, and simultaneous assessment of both relative (CCI) and absolute (AMCA) antagonist behavior strengthen the interpretability of the findings and provide a mechanistically oriented framework for understanding training-induced adaptations in older adults.

## Conclusions

The present study shows that both motor imagery training (MIT) and conventional strength training (CST) are associated with significant improvements in maximal elbow flexion force in older adults and reductions in relative agonist–antagonist co-contraction (CCI), without requiring significant changes in absolute antagonist muscle co-activation (AMCA). Exploratory within-subject analyses revealed that changes in maximal force were significantly associated with changes in CCI but not with changes in AMCA. In addition, exploratory agonist EMG analysis further indicated greater post-intervention agonist activation in both MIT and CST groups compared with control. Together, these findings are consistent with the possibility that strength gains in aging may be associated, at least in part, with improvements in relative coordination efficiency rather than suppression of antagonist activity alone, while modest changes in antagonist activation cannot be excluded.

Importantly, the dissociation between relative coordination (CCI) and absolute antagonist behavior (AMCA) further suggests that distinct dimensions of neuromuscular control may be differentially modifiable through behavioral intervention. MIT and CST were associated with similar functional outcomes, although the underlying mechanisms may differ. However, in the absence of direct neurophysiological measures, these mechanistic interpretations should be considered inferential, underscoring the adaptability of central motor control mechanisms in later life.

From a clinical perspective, these results support a mechanism-informed approach to upper-limb rehabilitation in older adults. Interventions targeting coordination efficiency and central motor drive may contribute to improvements force production, even when reductions in antagonist muscle activation are not observed. Motor imagery training may therefore represent a viable adjunct or alternative to resistance training for individuals with limited tolerance for physical loading, while conventional strength training may provide additional benefits when peripheral adaptation is desired.

Collectively, these findings contribute to a more nuanced understanding of strength adaptation in aging and provide a framework for tailoring rehabilitation strategies based on the specific neuromuscular mechanisms most amenable to modification.

## Data Availability

The raw data supporting the conclusions of this article will be made available by the authors, without undue reservation.
